# Meta-Analysis of Age, Sex, and Race Disparities in the Era of Contemporary Urothelial Carcinoma Treatment

**DOI:** 10.3390/cancers16193338

**Published:** 2024-09-29

**Authors:** Adam Barsouk, Omar Elghawy, Austin Yang, Jonathan H. Sussman, Ronac Mamtani, Lin Mei

**Affiliations:** 1 Abramson Cancer Center, Hospital of the University of Pennsylvania, 3400 Spruce Street, Philadelphia, PA 19104, USA; 2 Perelman School of Medicine, University of Pennsylvania, 3400 Civic Center Boulevard, Philadelphia, PA 19104, USA; 3 Graduate Group in Genomics and Computational Biology, Perelman School of Medicine, University of Pennsylvania, Philadelphia, PA 19104, USA

**Keywords:** urothelial cancer, disparities, race, sex, age, survival, immunotherapy, enfortumab, targeted therapy

## Abstract

**Simple Summary:**

Inclusion of Black patients and other racial minorities has been limited (<2%) in urothelial carcinoma clinical trials. On meta-analysis of all recent ICIs, ADCs and targeted therapy phase III trials in UC (*n* = 17), women had inferior OS to men (RR 0.89; *p* = 0.04) on investigational agents. No OS differences by race or age were observed on meta-analysis.

**Abstract:**

Background: Urothelial carcinoma (UC) is one of the most common cancers diagnosed worldwide. However, minority populations, such as female, elder, and Black patients, may have disparate outcomes and are commonly neglected in randomized prospective trials. This review aims to study the relationship between age, sex, and race on urothelial cancer prognosis, particularly focusing on contemporary therapy and its effect on overall survival. Methods: Phase III prospective trials since 2016 of immune checkpoint inhibitors, antibody-drug conjugates, or targeted therapies in urothelial carcinoma were identified from PubMed. Trials that did not report on survival by race, sex, or age distribution were excluded, and remaining trials (*n* = 17) were compared by subgroup. Results: Women were reported to have inferior OS on investigational agents compared to men in 9/17 trials. In a meta-analysis, women had inferior OS to men (OR 0.89 [95% CI: 0.78–0.99]; *p* = 0.04). Asian/Pacific Islander patients had inferior outcomes to White patients on investigational agents in 3/5 trials. In a meta-analysis, OS was not significant by race (OR 1.18 [0.90–1.46], *p* = 0.38). Black patients composed <2% of all trial patients, and no subgroup data were reported. Both 65 (*n* = 7) and 75 (*n* = 2) were reported as age cut-offs in trial subgroups, and survival data were mixed. Conclusions: Women in UC trials may have inferior survival outcomes to men. Racial diversity was poor and thus limited any conclusions on survival disparities.

## 1. Introduction

Urothelial carcinoma (UC) is the sixth most common cancer diagnosis worldwide and in the United States [1,2]. In total, 90% of urothelial cancer diagnoses are made in those 55 years of age and older, and the disease is four times more common in men than women and around double as likely in non-Hispanic Whites as compared to Black or Asian patients [3]. Despite recent approvals of immune checkpoint inhibitors (ICI), antibody-drug conjugates (ADC), and targeted therapy, the prognosis for advanced UC (aUC) remains poor, with a 5-year survival rate of under 5% [4]. Moreover, women and non-White races constitute a minority of UC patients, and limited retrospective data suggest that these groups may receive a diagnosis at later stages and suffer worse survival irrespective of stage, although prospective data are lacking [5,6,7,8,9]. Likewise, while novel agents and approaches have allowed for older and poorer performance status patients to receive local and systemic therapy in the real world, older patients continue to receive fewer treatments and suffer poorer survival per limited retrospective studies [7,10,11]. In particular, there is a paucity of data on how the introduction of novel ICI, ADC, and targeted therapy agents for aUC has affected disparities in survival by age, sex, and race.

We present a meta-analysis of phase III prospective trials of ICI, ADC, and targeted agents since 2016 by reported subgroups of race, sex, and age to elucidate the impact that these patient factors may have on survival in aUC.

## 2. Methods

### Study Population

**Figure 1:**
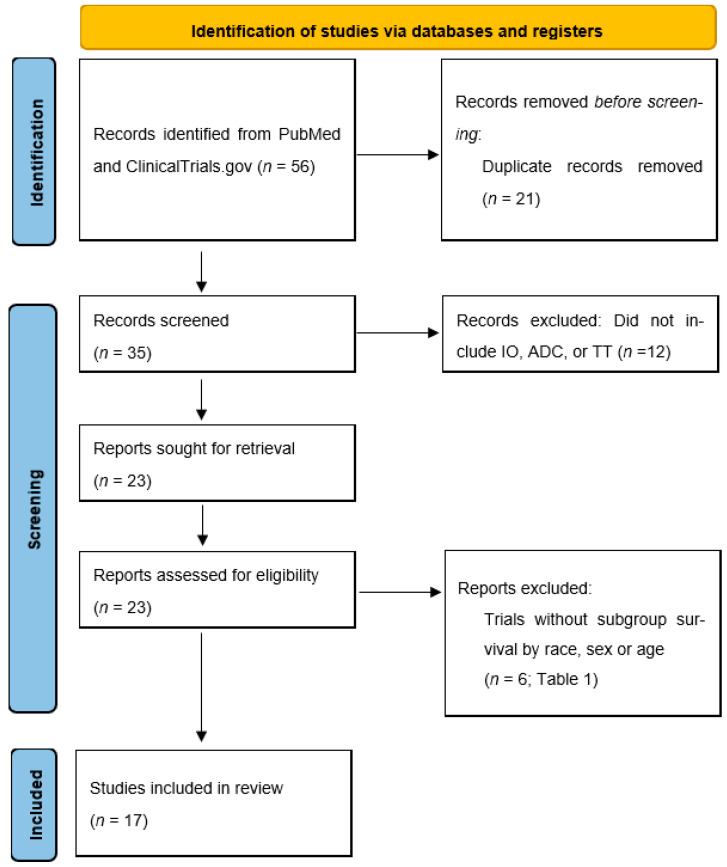
Review methodology PRISMA diagram. A systematic review of PubMed and clinicaltrials.gov was performed in August 2024 by using the following search terms: “urothelial” AND (“cancer” OR “carcinoma” OR “tumour” OR “tumor” OR “neoplasm” OR “malignancy” OR “mass”) AND (“trials”). Results were filtered to include only phase II–III trials of IO, ADC, or targeted therapies. Trials without demographic data on race, sex, or age distribution were excluded. Remaining trials were then separated based on whether subgroup survival by race, sex, or age was reported.

We conducted a systematic review using PubMed and ClinicalTrials.gov of prospective phase III trials evaluating ICIs (pembrolizumab, avelumab, atezolizumab, nivolumab, durvalumab + tremilimimab), ADCs (enfortumab vedotin, sacituzumab govitecan), and targeted therapies (erdafitinib) since 2016. The analysis was conducted in August 2024. We used the following Boolean strategy in our search: A combination of the following search terms was used: “urothelial” AND (“cancer” OR “carcinoma” OR “tumour” OR “tumor” OR “neoplasm” OR “malignancy” OR “mass”) AND (“trials”) AND (“immunotherapy”) or (“antibody drug conjugate”) (ADC) or (“erdaftinib”) and filtering from 2016 to present. The immunotherapy search yielded 112 results, while the ADC search yielded 12 results; targeted therapy yielded 5 results. Those results were filtered to include only phase III trials specific to UC, yielding 23 results. Trials were evaluated for inclusion of baseline demographics, namely age, sex, and race, and the subgroup analyses were evaluated for survival endpoints by age, sex, and race. Two authors independently conducted the screening, and for any paper that was identified by one author but not the other, a third author was consulted to determine if the identified report met inclusion criteria, which was then taken as the final decision (Figure 1). A meta-analysis of overall survival odds ratio for investigation agents was performed with a weighted-mean difference analysis for men vs. women, White vs. Asian/Pacific Islander patients, and <65 vs. >65-year-old patients. A *p* value < 0.05 was considered significant. R v4.4.0 was used for statistical analysis (Foundation for Statistical Computing, Vienna, Austria). The meta for R package was used for the evaluation of the meta-analysis’ publication bias and the study’s heterogeneity.

## 3. Results

### 3.1. Sex

**Figure 2:**
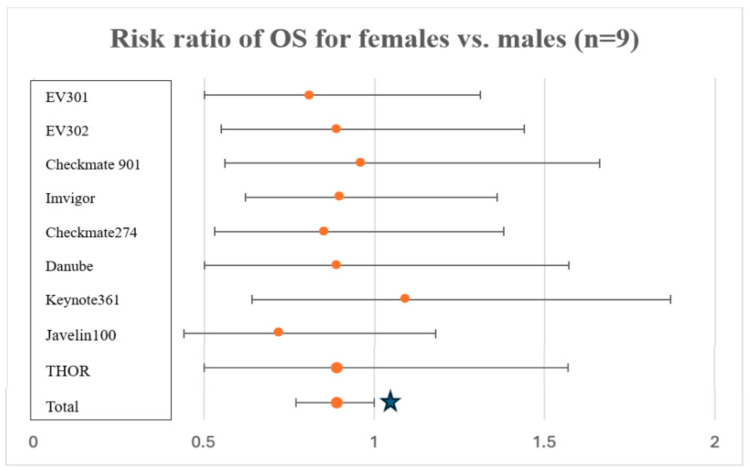
Investigational agent OS forest plot (females vs. males). Risk ratio of OS for females vs. males in investigational agent arm for all trials that reported subgroup analysis by sex (*n* = 9). In a cumulative meta-analysis, women were found to have significantly poorer OS as compared to men (RR 0.89 [0.78–0.99], *p* = 0.04, star for significance).

**Figure 3:**
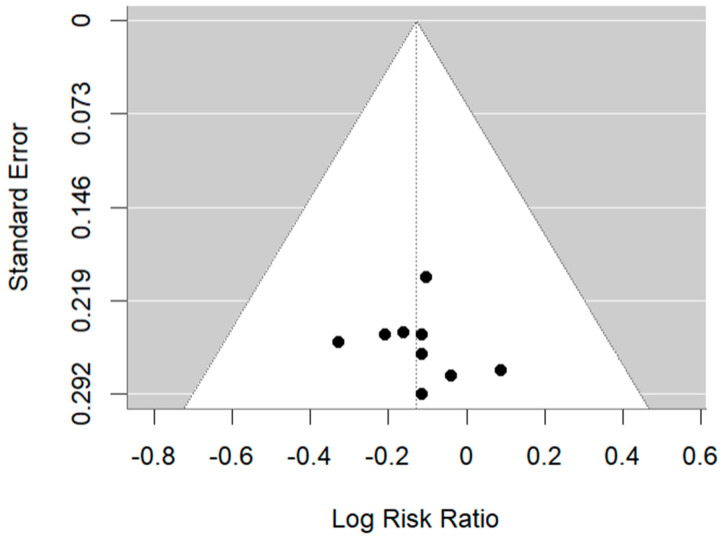
Funnel diagram. Funnel diagram for clinical trials reporting OS subgroup by sex. Eggers test was *p* = 0.80, indicating no evidence of publication bias.

All 17 prospective trials evaluated reported sex distribution (*n* = 13,472), and they were all composed of 70–80% male patients. Nine of the seventeen trials (53%) reported a subgroup analysis by sex for OS, of which five (56%) found that women did not achieve an OS benefit from the investigational agent, while men did (i.e., inferior outcomes for women vs. men; Table 1). In a meta-analysis of the investigational arm of the nine trials (*n* = 3233; 24%), women were found to have a significantly lower odds ratio of OS compared to men (OR 0.89 [95% CI: 0.78–0.99]; *p* = 0.04; Figure 2). Eggers test was *p* = 0.80, indicating no evidence of publication bias (Figure 3). The I^2^ value is 0 in this case, which indicates that there is no significant heterogeneity between trial results.

### 3.2. Race

Of the 17 trials evaluated (*n* = 13,472), 9 reported racial distribution beyond “White vs. other” (53%). In these nine trials, Black patients never exceeded 2% of the overall study population. Asian/Pacific Islander (API) patients composed 7–33% of the study populations where reported. Five of the seventeen trials analyzed (29%) reported subgroup OS analysis by race, of which three trials (60%) found that API patients did not achieve a survival benefit on the investigational agent, while White patients did (i.e., inferior outcome for API vs. White patients; Table 1). On a meta-analysis of the investigational arm of the five trials (*n* = 2146), the total OR was 1.18 [0.90–1.46], *p* = 0.38 for API patients vs. White patients. No trial (0%) reported an OS subgroup for Black patients.

### 3.3. Age

Of the 17 trials evaluated, 9 (53%) reported a subgroup analysis for OS by age, with a cut-off at 65 (*n* = 7 trials) or 75 years old (*n* = 2 trials). Four of the nine trials (44%) found that older patients did not achieve superior survival with the investigational agent, while younger patients did (i.e., inferior outcomes for older vs. younger patients), while two trials (22%) reported patients <65 years had inferior survival on the investigational agent as compared to older patients (i.e., superior outcomes for older vs. younger patients) (Table 1). In a meta-analysis of the seven trials that reported subgroup OS analysis by age >65 vs. <65 (*n* = 3144), the OS odds ratio was 1.11 [95% CI: 0.92–1.32]; *p* = 0.47 for older vs. younger patients on the investigational agent. Only one trial reported subgroup analysis by age > 75 (EV301), with a HR of 0.84 [0.46–1.53].

## 4. Discussion

### 4.1. Disparity in Survival by Sex

Our meta-analysis revealed that women were less likely to derive a survival benefit from investigational agents than men, and in fact showed an inferior odds ratio for OS when compared to men. These findings are in keeping with retrospective studies from the US and abroad that demonstrate significant disparities in survival for women compared to men. A study of excess hazard in the SEER database in the US from 1990 to 2005 concluded 70% of excess hazard of death for women could not be explained by age or tumor characteristics [7]. A study of the Swedish Urinary Bladder Cancer Register (*n* = 36,344) likewise found that women had a higher bladder cancer mortality (adjusted HR, 1.15; 95% confidence interval, 1.08–1.23), although the disparity was limited only to those patients with muscle-invasive tumors (adjusted HR, 1.24; [1.14–1.34]) [12,13]. In a study of a Japanese population registry from 1993 to 2006, women showed higher risk of death in localized UC (HR 1.29, [1.05–1.57]; *p* = 0.0145) and locally advanced UC (HR 1.32, [1.15–1.52]; *p* = 0.0001), but no difference in the metastatic UC (HR 1.04, [0.87–1.25]; *p* = 0.6555) [14]. Of note, a retrospective study of the SEER database (2004–2016) that focused on variant histology bladder cancer (VHBC), which is poorly represented or excluded from most UC studies, likewise demonstrated a more advanced stage diagnosis for women (OR = 1.55, *p* = 0.0001) and greater five-year mortality independent of stage (HR = 1.25, *p* = 0.02) [15,16].

Men are vastly more likely to receive bladder cancer than women, with biological sex differences accounting for much of this disparity [5]. In murine bladder cancer models, androgen has been found to create a microenvironment that promotes tumor growth by inhibiting thrombospondin-1, an antiangiogenic factor [17]. Reductions in androgen levels in both male and female mice prevented N-butyl-N-(4-hydroxybutyl)nitrosamine-induced bladder cancer development, attributable to the suppression of cell proliferation and increase in apoptosis [18]. In contrast, estrogen promotes stronger immune responses, particularly in response to carcinogens, which has a tumor-suppressive effect [19]. These factors may contribute to the higher incidence of bladder cancer in men.

In women, bladder cancer often presents at higher stages, partly due to a longer time from initial signs such as hematuria to bladder cancer diagnosis [5]. Women are two times less likely than men to go to doctors for hematuria, two times more likely to get diagnosed with urinary tract infection when presenting with hematuria, and also less likely to receive abdominal or pelvic imaging in response to hematuria, causing delays in diagnosis [5,19]. In the study conducted by Scosyrev et al., 13% of White men and 19% of Black men diagnosed with bladder cancer were diagnosed with stage 3 or 4 bladder cancer, while 15% of White and 29% of Black women diagnosed with bladder cancer were diagnosed with stage 3 or 4 bladder cancer [7]. Women are more likely to have locally advanced cancer, resulting in a worse prognosis (23.9% 5-year survival versus 87.7% 5-year survival of women with localized cancers) [13,14]. Women exhibit worse outcomes after radical cystectomy, and it has been speculated that inherent anatomical differences are partially responsible [14,15,16]. Women also have a higher risk of recurrence after BCG immunotherapy, further lowering OS in comparison to men [6]. Across tumor types, women have demonstrated poorer tolerability of immunotherapy, leading to greater rates of discontinuation, as well as poorer efficacy regardless of tolerability. This discrepancy is believed to be multifactorial, with similar etiology to autoimmune disease, e.g., X-chromosome chimerism, antigens from pregnancy, and hormonal effects [20]. Tumors in women have also been shown to demonstrate more successful escape mechanisms of immune surveillance [21]. While these effects have been well characterized for ICIs, their impact on the efficacy and tolerability of ADCs like enfortumab or sacituzumab, which also rely on the body’s immune response, remains to be elucidated. Recently, some advances have been made into understanding the bladder microbiome in men and women [5]. Overall, women are more likely to be diagnosed at more advanced stages and show worse responses to surgical and pharmacologic treatment.

### 4.2. Disparity in Survival by Race

Our meta-analysis did not reveal any significant survival difference between White and Asian/Pacific Islander patients; however, no conclusions could be drawn for Black Americans due to very limited trial inclusion (<2% of all participants when reported). Furthermore, only 29% of trials evaluated reported any subgroups by race, thus limiting the power of our meta-analysis. Evaluation of racial disparities in outcomes has been limited in prospective trials in UC. A systematic review of 544 clinical trials from 1970 to 2020 in UC revealed only 4.4% of trials reported race, and in those trials, Black (2–8%) and Hispanic patients (2–5%) were underrepresented [12].

Despite the dearth of prospective data, several retrospective studies have suggested inferior outcomes for Black Americans based on publicly available population data. The Hasan et al. study of the National Cancer Database from 2004 to 2016 identified over 400,000 UC patients across stages, and concluded that Black race, Hispanic ethnicity, and female sex were all independently associated with decreased likelihood of receiving treatment, regardless of disease stage [8]. The Fang et al. study, which analyzed the SEER database from 2010 to 2015, identified over 100,000 UC patients, around 6% of whom were Black and 0.3% American Indian/Alaska Native (AIAN) [9]. They found that both Black and AIAN patients had poorer OS compared to White or Asian/Pacific Islander (API) patients, even when controlled for other factors with a predictive nomogram on multivariable analysis. An additional study of the SEER database from 1990 to 2005 estimated the excess hazard of death for Black and female patients due to age and tumor characteristics and concluded that 30–50% of excess hazard of death could not be explained for Black patients [7].

With the racial demographic data available, Black patients exhibit worse prognoses in comparison to White and Asian patients and are more likely to be diagnosed at later stages of bladder cancer [9]. This has been attributed in part to reduced access to healthcare and distrust of the medical system [7,8,22,23]. Black patients are more likely to have muscle invasion (OR = 1.2), locally advanced disease (OR = 1.49), and metastatic disease (OR = 1.6) [24]. In addition, Black patients are less likely to receive “optimal treatment,” defined based on recommendations by the National Comprehensive Cancer Network, especially in late-stage tumors (OR = 0.51 in muscle invasive disease) [8,24]. Black patients are less likely to receive surgical treatments, often due to lack of access to tertiary care centers [7,9,25,26,27]. The delays in diagnosis and multimodal therapy result in these racial disparities in prognosis [25,26]. Biological differences may likewise contribute disparities; for instance, Black Americans have higher proportions of non-urothelial histologies such as squamous cells, which have been attributed to greater risk of occupational exposure and schistosomiasis [28]. Further studies are warranted to quantify the proportion of disparity that can be accounted for by biology versus structural racism.

### 4.3. Disparity in Survival by Age

Our meta-analysis revealed conflicting findings on survival disparities by age, with 4/9 trials reporting poorer OS for older adults, and 2/9 trials reporting superior OS for older adults. The analysis was further limited by variable subgroup age cut-offs, with 7/9 reporting data for >65 years and 2/9 reporting data for >75. These findings stand in contrast to the retrospective literature. A retrospective analysis of 117,275 UC patients in the US Surveillance, Epidemiology, and End Results (SEER) database from 2004 to 2015 found that patients diagnosed with UC >75 years old had inferior overall survival (OS) compared to those <54 (hazard ratio [HR] = 5.36, as well as inferior bladder-cancer-specific OS) [10]. At 5 years, the overall cumulative mortality of bladder cancer patients over >75 years of age was 55.0% versus 15.7% for patients ≤54 years of age [10]. Older patients are also often ineligible for surgery and chemotherapy due to preexisting conditions [29,30]. Organ dysfunction limits the dosage of chemotherapy drugs, worsening their efficacy [30]. These restrictions present an opportunity for immunotherapy drugs which have lower rates of adverse effects while still remaining effective. Atezolizumab as a first-line treatment in platinum-ineligible patients aged 51–92 with a median age of 73 years saw a 23% objective response rate (ORR) with a lower rate of adverse effects compared to conventional chemotherapy [31]. Combining immuno- and chemotherapy also improves outcomes, as seen with durvalumab, which, as a second-line treatment following chemotherapy, saw an ORR of 17.7% and a low rate of adverse events [32]. Although older people have weaker immune systems, patients aged >65 years demonstrated immunotherapy efficacy comparable to younger patients [31,33,34]. Studies of PD-L1 inhibitors for other malignancies, including metastatic melanoma, metastatic non-small cell lung cancer, and metastatic renal cell carcinoma, also concluded that their efficacy does not significantly vary with age [34]. These findings highlight the importance of multimodal therapy in treating older patients.

### 4.4. Limitations

Our meta-analysis was severely limited by the lack of diversity in race among trial populations, particularly with poor inclusion of Black Americans (<2%), as well as lack of reporting on data by subgroups (only 9/17 trials reported OS subgroups for age, sex, or race). This phenomenon is well characterized in the aUC literature. A systematic review of 544 clinical trials found only 4.4% of trials report racial demographic data [12]. Even when reported, Black and Hispanic patients were underrepresented, causing trials to have insufficient Black patients to include in the subgroup analysis [12]. Non-standard age cutoffs also limit data analysis, making it difficult to draw broad conclusions about whether age affects ICI or ADC efficacy [35].

## 5. Conclusions

Our analysis demonstrates that recent prospective trials have included inadequate racial diversity and have failed to regularly conduct subgroup survival analyses by age, sex, and race. Among the studies that reported survival by subgroup, half found that women had inferior outcomes to men (which was re-demonstrated in the meta-analysis), and around a third of studies found that Asians and Pacific Islanders had inferior outcomes to White patients (Black patients were too few to be compared). The subgroup analysis on age was mixed, with some trials reporting poorer outcomes for older patients and others finding poorer outcomes for younger patients. Retrospective trials confirmed these disparities, reporting significant evidence across countries that women had poorer outcomes to men, even when adjusted for other factors. Retrospective studies also revealed significantly poorer outcomes for Black, Hispanic, and AIAN patients in the US. Data for patients of older age was more limited, although limited studies suggested poorer survival and less likelihood to receive treatment. ICIs and ADCs represent a new treatment modality for late-stage UC that is less straining on the body than chemotherapy, and prospective trials have demonstrated comparable efficacy in both younger and older populations. These novel agents may provide a means of lessening survival disparities by race, sex, and age, although greater inclusion of diverse populations in trials is paramount.

## Figures and Tables

**Table 1:** Prospective trials.

Trial	Agent	Indication	% Female	OS Female	% Black	% Asian	OS Race	% >75	OS >75
EV-301	EV	1L Cis-ineligible	22.7	NSB *	NR	NR	NR	17.3	NSB *
EV-302	Pembro + EV	1L Cis-ineligible	22.2	ND vs. men	0.7	22.4	NR	23.1	ND
Checkmate 901	Nivo+ GC	1L	22.4	NSB *	0	24.7	All races NSB	11.2	NSB *
KEYNOTE 052	Pembro	1L Cis-ineligible	23	NR	2.2	7	NR	49	ND
IMVigor211	Atezo	2L	30	NR	0.3	12.7	NR	NR	NR
IMVigor 130	Atezo + GC	1L	25	NSB *	1	22.6	Asian: NSB *	NR	<65 NSB
Javelin Bladder 100	Avelumab	1L maintenance	24	NSB *	0.8	17.9	Asian/Other: NSB *	NR	<65 NSB
DANUBE	Durvalumab +/− Tremilimumab	1L	26	NB all	0.6	21.5	NB all	NR	NB all
KEYNOTE 361	Pembro +/− chemo	1L	24.5	NB all	0.8	17.9	NR	NR	NR
THOR	Erdafitinib	2L *FGFR2/3*-mt	29.4	NSB *	0	27.2	NR	NR	NR
Checkmate 274	Nivo	Adjuvant	24.9	NSB *	0.6	22.7	Asian: NSB *	17.3	NSB *

All other trials did not report subgroup analyses by age, race, or sex. Abbreviations: OS: Overall Survival, NB: no benefit, NR: not reported, ND: no difference, EV: enfortumab vedotin, NSB: No Survival Benefit, GC: Gemcitabine+Cisplatin, EV: enfortumab vedotin. * In contrast to other patient cohorts who did achieve significant survival benefits.

## Data Availability

No new data were created or analyzed in this study. Data sharing is not applicable.
